# Case Report and Minireview of the Literature on Blunt Azygos Injury

**DOI:** 10.14789/jmj.JMJ22-0010-CR

**Published:** 2022-07-14

**Authors:** KEN-ICHI MURAMATSU, KEI JITSUIKI, SHUNKI HIRAYAMA, YOUICHI YANAGAWA

**Affiliations:** 1 Department of Acute Critical Care Medicine, Shizuoka Hospital, Juntendo University, Shizuoka, Japan; 1 Department of Acute Critical Care Medicine, Shizuoka Hospital, Juntendo University, Shizuoka, Japan; 2 Department of Thoracic Surgery, Shizuoka Hospital, Juntendo University, Shizuoka, Japan; 2 Department of Thoracic Surgery, Shizuoka Hospital, Juntendo University, Shizuoka, Japan

**Keywords:** trauma, shock, azygos injury, outcome

## Abstract

Azygos vein injury seems to be an uncommon cause of hemothorax and hemomediastinum; however, this injury is potentially fatal. We report a fatal case of blunt azygos injury and a PubMed search was undertaken to identify English articles from 1989 to 2022 using the key words “azygos”, “injury” and “blunt”. We found 28 articles about blunt azygos injury and 39 patients including the present case (average 41.2 years [range: 18-81 years]; male, n=20; female, n=19). The other variables were as follows: right hemothorax (n=32); unstable circulation on arrival (n=32); and survival (n=19; unknown, n=2). These cases were divided into two groups based on the outcome: the survival group and the fatal group. There were no significant differences with regard to the year of the report, age, sex, rate of right rib fracture, rate of preoperative computed tomography (CT) examination, rate of associated injury, and rate of operation. The rate of shock on arrival in the survival group was significantly lower than that in the fatal group. The rate of azygos arch injury in the survival group was significantly greater than that in the fatal group. The emergency physician must consider azygos vein injury as a possible cause of right hemothorax when a patient with blunt chest trauma presents persistent hypotension.

## Introduction

The azygos vein is located on the right side of the vertebral column and penetrates from the retroperitoneum through the diaphragm to join the superior vena cava at the T4 level^1)^. Fracture- dislocation of the mid-thoracic spine or ribs, as a result of blunt thoracic trauma, can tear the azygos vein^1)^. The vein can also be torn, in the absence of skeletal injuries, by horizontal acceleration/deceleration forces^1)^. Most reports of blunt trauma to the azygos vein in the relevant literature are related to motor vehicle collisions^1)^. Patients frequently present with shock-like symptoms and expanding hemothorax, necessitating prompt surgical repair^1-28)^. Azygos vein injury seems to be an uncommon cause of hemothorax and hemomediastinum; however, this injury is potentially fatal. We herein report a fatal case of blunt azygos injury and a review of the relevant literature. The protocol of this retrospective study was approved by Juntendo Shizuoka Hospital review board (approval number: 298). We obtained oral informed consent from the bereaved.

## Case presentation

**Figure 1:**
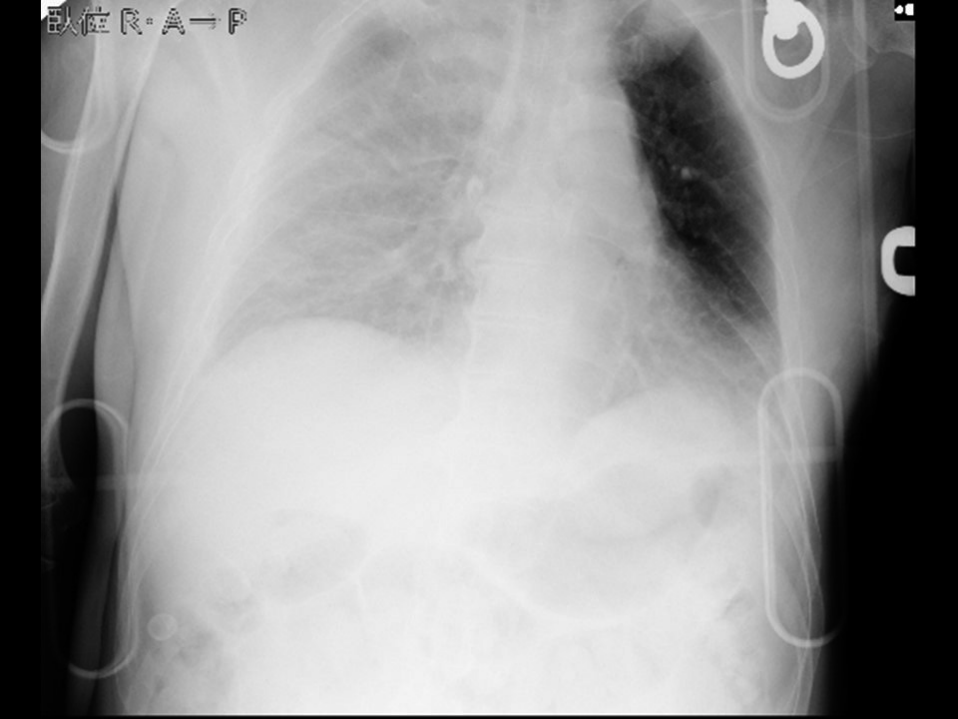
Chest X-ray on arrival The X-ray suggested right hemothorax.

**Figure 2:**
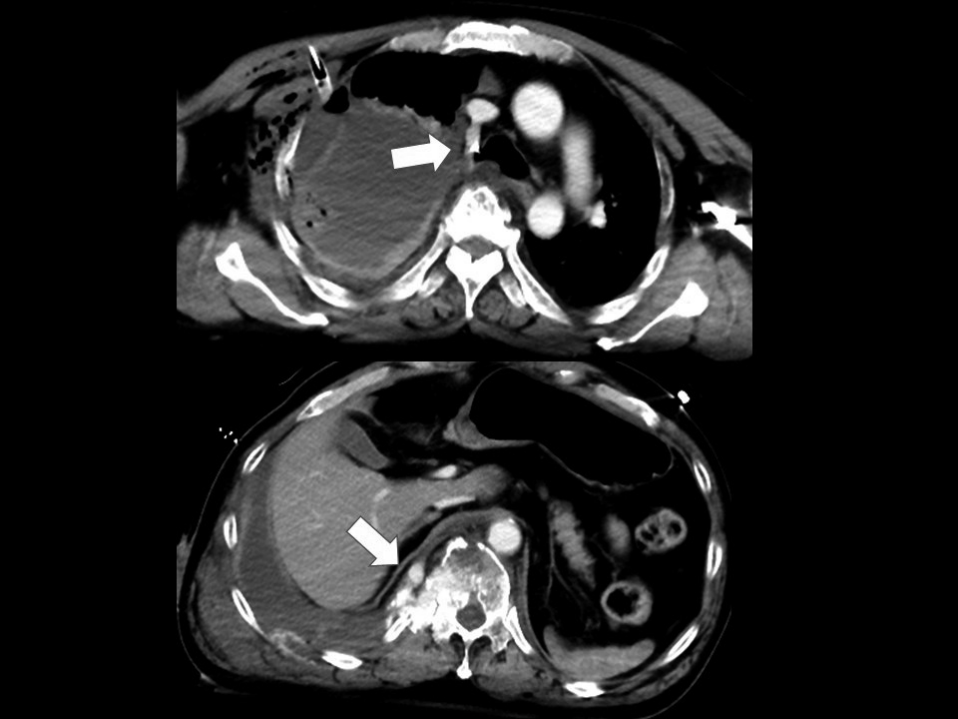
Enhanced chest computed tomography after tentative thoracotomy Bleeding from the azygos arch was controlled (upper arrow) but hemorrhaging from the inferior azygos vein near the thoracic vertebral fracture remained (lower arrow).

A 63-year-old man fell from a 2^nd^ floor veranda while leaning over a banister trying to catch a ladder. When emergency medical technicians checked him, he was in shock state with consciousness disturbance; thus, he was transported to our emergency room (ER) by ambulance within 20 minutes. He had a medical history of diabetes mellitus and colon cancer. On arrival, his vital signs were as follows: Glasgow Coma Scale, E4V3M6; blood pressure, 75/- mmHg; heart rate, 140 beats per minute; respiratory rate, 30 breaths per minute and percutaneous saturation, 98% under 10 L per minute of oxygen. A physical examination revealed a head contusion and weakness of the right respiratory sound. The chest roentgenography showed decreased radiolucency in the right lung field (Figure 1), suggesting right hemothorax. Focus assessment of sonography for trauma also showed fluid collection, which was limited to the right thoracic cavity. Initially, he underwent immediate massive transfusion without cross-matching and tracheal intubation following right thoracostomy, which drained over 1 L of hemorrhaging. As his blood pressure did not respond to massive transfusion, right thoracotomy was tentatively performed by young emergency physicians in order to pack gauze and achieve hemostasis around the pulmonary hilus, where blood was emerging without a hilar clamp, while the patient was in the supine position. However, his unstable circulation deteriorated. After closing the thoracotomy, he was moved to the computed tomography (CT) room and CT revealed hemorrhaging from the inferior azygos vein near a thoracic vertebral fracture (Figure 2) and right subdural hematoma. He experienced cardiac arrest after returning to the ER. A thoracic surgeon standing by at home attended the ER and explored the right thoracic cavity by opening the thoracotomy. The surgeon recognized an azygos arch injury and achieved hemostasis by gauze packing. The surgeon also performed manual compression at the hemorrhaging site of the inferior azygos vein, and transfusion was continued. However, a return of spontaneous circulation was not obtained due to hemorrhaging associated with the trauma itself and the operative incision site due to the patient's bleeding tendency.

## Review and analysis of the relevant literature

A PubMed search was undertaken to identify English articles from 1989 to 2022 using the key words “azygos”, “injury” and “blunt”. We found 28 articles about blunt azygos injury^1-28)^. We summarized these cases, including the present case, in Table 1. We also added the report by Wall et al. into Table 1 as a supplement, which described the treatment of the largest series of penetrating azygos injury cases in the relevant literature^29)^. There were 39 cases of the blunt azygos injury (average age, 41.2 years [range: 18-81 years]; male, n=20; female, n=19). The mechanisms of injury were as follows: traffic accident (n=29); fall (n=4), falling object (n=1), assault (n=1), sports (n=1), chest compression for cardiac arrest (n=1), and unknown (n=2). The other variables were as follows: right hemothorax (n=32; unknown, n=1); unstable circulation on arrival (n=32; unknown, n=1); right rib fracture, (n=20; unknown, n=5); preoperative CT examination (n=12), associated injury (n=30; unknown, n=1); surgical operation (n=36; unknown, n=2); and survival (n=19; unknown, n=2). While, a delayed appearance of right hemothorax after blunt chest trauma due to traumatic azygous vein injury, likely from rupture of a pseudoaneurysm, was observed. Thus, the diagnosis of azygous vein injury without initial hemothorax can be hampered in extremely rare case^16)^. These cases were divided into two groups: the survival group (n=19), which included cases in which the outcome was survival; and the fatal group (n=18), which included cases who died. The characteristics of the cases were compared between the two groups, including the year of the report, age, sex, rate of shock on arrival, rate of right rib fracture, rate of azygos arch injury, rate of preoperative CT, rate of associated injury, and rate of operation. The chi-squared test, median test or non-paired Student's *t*-test were used for the statistical analyses. P values of <0.05 were considered to indicate statistical significance. The results of the analysis are shown in Table 2. There were no statistically significant differences with regard to the year of the report, age, sex, rate of right rib fracture, rate of preoperative CT examination, rate of associated injury and rate of operation. The rate of shock on arrival in the survival group was significantly lower than that in the fatal group, and the rate of azygos arch injury in the survival group was significantly greater than that in the fatal group.

**Table 1:** Summary of case of blunt azygos injury

No	Name	Refe-rence	year	age	sex	mechanism	right hemo- thorax	shock	rib fracture	site	CT	associated injury	Ope- ration	Sur- vival
1	Li	1	2022	81	female	traffic accident	yes	yes	yes	arch	no	diaphragma, liver, omentum, pelvis	yes	yes
2	Li	1	2022	38	male	traffic accident	yes	yes	yes	arch	no	Th3, L4, pelvis	yes	yes
3	DeMaio	2	2021	28	female	traffic accident	yes	no	yes	arch	yes	Th4,5, sternum	yes	yes
4	Laohathai	3	2019	33	female	traffic accident	yes	no	no	arch	no	radius	yes	yes
5	Papadomanolakis	4	2016	28	female	traffic accident	yes	yes	no	?	no	liver, femur	yes	no
6	Papadomanolakis	4	2016	50	male	traffic accident	yes	yes	yes	?	no	Th, liver, pelvis	yes	no
7	Papadomanolakis	4	2016	28	male	traffic accident	yes	yes	yes	?	no	femur	yes	no
8	Papadomanolakis	4	2016	35	male	traffic accident	yes	yes	yes	?	no	liver	yes	no
9	Papadomanolakis	4	2016	41	male	fall	yes	yes	yes	?	no	liver, spleen	yes	no
10	Papadomanolakis	4	2016	20	male	traffic accident	yes	yes	yes	?	no	liver, kidney, femur	yes	no
11	Papadomanolakis	4	2016	65	female	traffic accident	yes	yes	yes	?	no	pelvis	yes	no
12	Yang	5	2014	52	female	chest compression	yes	yes	yes	?	yes	none	yes	no
13	Mohajeri	6	2014	45	male	sport	no	no	no	arch	yes	none	no	yes
14	Haq	7	2014	52	male	traffic accident	?	?	?	arch	yes	?	?	?
15	Cao	8	2012	60	male	falling object	yes	yes	yes	arch	yes	lumbar, lower extremity	yes	yes
16	Juraszyński	9	2010	70	female	blunt	no	no	no	SVC junction	yes	none	yes	yes
17	Endara	10	2010	21	male	traffic accident	yes	yes	?	SVC junction	no	spleen	yes	no
18	Drac	11	2007	22	male	traffic accident	yes	yes	yes	?	yes	head, spleen	yes	no
19	Kamiyoshihara	12	2007	71	female	traffic accident	yes	?	yes	arch	yes	kidney, leg	yes	yes
20	Nguyen	13	2006	21	male	traffic accident	yes	yes	?	Th4	no	femur, descending aorta	yes	yes
21	Bowles	14	2000	21	male	traffic accident	yes	yes	?	arch	yes	none	yes	yes
22	Sharma	15	1999	75	female	traffic accident	yes	yes	yes	arch	no	liver, head, pelvis	yes	yes
23	Sugimoto	16	1998	44	male	traffic accident	no	yes	no	arch	no	abdomen	yes	no
24	Cagini	17	1998	18	female	traffic accident	yes	yes	no	arch	no	omentum	yes	yes
25	No author	18	1996	51	female	blunt	yes	no	no	hemidiaphragm	yes	none	?	?
26	Butler	19	1995	23	male	traffic accident	yes	yes	yes	arch	no	head, Th3,4, spleen, tibia	yes	yes
27	Jain	20	1994	51	female	assault	yes	yes	no	Above diaphragm	no	none	yes	no
28	Inoue	21	1993	41	female	traffic accident	yes	yes	no	SVC junction	no	none	yes	yes
29	Walsh	22	1992	41	male	fall	yes	yes	no	SVC junction	no	none	yes	no
30	Thurman	23	1992	19	male	traffic accident	yes	yes	?	arch	no	head, ankle	yes	yes
31	Shkrum	24	1991	23	male	fall	no	yes	no	Th5	yes	Th, head	yes	no
32	Shkrum	24	1991	39	female	traffic accident	no	yes	no	Th4	no	Th, head, liver, spinal cord	yes	no
33	Shkrum	24	1991	48	female	traffic accident	yes	yes	yes	Th4	no	Th, liver, spleen	yes	no
34	Shkrum	24	1991	24	female	traffic accident	no	yes	no	Th4-5	no	pelvis	yes	no
35	Baldwin	25	1984	28	female	traffic accident	yes	yes	yes	SVC junction	no	abdomen, lower extremities	yes	yes
36	Sherani	26	1986	25	male	traffic accident	yes	yes	no	Th4-5	no	head, abdomen, lower extremities	yes	yes
37	Coates	27	1987	63	female	traffic accident	yes	yes	yes	SVC junction	no	head, abdomen, lower extremities	yes	yes
38	Snyder	28	1989	52	female	traffic accident	yes	yes	yes	Inferior SVC junction	no	spine	yes	yes
39	Present case		2021	63	male	fall	yes	yes	yes	arch, Th12	yes	head	yes	no
														
	Wall	29	2006	?	?	gun shot 19, stab 3	?	?	?	?	?	Multiple	yes	mortality 36%

CT: computed tomography, Th: thracic spine, L: lumbar spine, SVC: superior vena cava

**Table 2:** Comparison between the survival and fatal groups

	Survival n = 19	Fatal n = 18	p value
Year of the report	2000	2012	0.24
Age	42.7 ± 21.3	38.6 + 14.3	0.77
Sex (male/female)	8/11	11/7	0.24
Shock (%)	14 (73)	18 (100)	0.01
Right rib fracture (%)	10/n=16 (62)	10/n=17 (58)	0.82
Arch (%)	12 (63)	1/n=9 (11)	0.006
CT (%)	6 (46)	4 (22)	0.52
Associate injury (%)	15 (78)	15 (83)	0.73
Operation (%)	18 (94)	18 (100)	0.24

CT; computed tomography

## Discussion

This review of cases of blunt azygos injury is the first report to suggest that shock on arrival and the location of azygos vein injury may have an influence on final outcome of the patient.

Shock on arrival in patients with blunt trauma suggests massive bleeding from injured sites and/or spinal cord injury, and previous reports have also demonstrated that shock on arrival is a poor prognostic factor^30, 31)^. Accordingly, ER physicians must consider azygos vein injury as a possible cause of right hemothorax in patients with blunt chest trauma who show persistent hypotension. The reason for the favorable outcome of azygos arch injury in comparison to other sites might be that it is easier to visually recognize the injured site. Usually, trauma patients are managed in supine position in the ER and tentative thoracotomy is also performed in the same position because subsequent tentative laparotomy might be required to explore abdominal injuries^32)^. The azygos arch was easily visually recognized in the supine position, however, other sites might be hidden by the pulmonary hilus, lung or diaphragm^33)^. In the present hemostasis at the site of the azygos arch injury was obtained by direct gauze packing; however, the packing at the inferior injury site of the azygos vein was insufficient.

This review of cases of blunt azygos injury failed to show that recent medical development has resulted in favorable outcomes. Recent surgeons are familiar with using preoperative radiological studies to perform a planned operation precisely, safely and less invasively. In contrast, experienced trauma surgeons can perform urgent surgical operations without radiological studies, with manual intraoperative exploration to identify the site of bleeding and apply hemostasis^34)^. Advanced Trauma Life Support^®^ (ATLS^®^) does not recommend that trauma patients with unstable circulation be moved to a CT room or for CT examination to be used to identify sites of bleeding^35)^. However, it is important for hemostasis to be immediately achieved at hemorrhaging sites in patients with unstable circulation. The number of patients with severe trauma has been decreasing year by year, and the number of experienced trauma surgeons in Japan has declined^36)^. The fact that the diagnostic studies included as part of the initial ATLS^®^ trauma survey are not well equipped to diagnose such a fatal vascular injury^37)^. In addition, recent studies showed the efficacy of evaluation using whole CT during resuscitation in the hybrid ER, for even trauma patients with unstable circulation, in order to detect sites of hemorrhaging and facilitate the immediate performance procedures to obtain hemostasis^38-41)^. Accordingly, to increase the survival rate of patients with fatal vascular injuries, such as blunt azygos injury, the early recognition of the site of hemorrhaging using CT and the immediate execution of surgical hemostasis in an appropriate position for modern surgeons (less experienced in the management of severe trauma) may be required, even when initial fluid resuscitation fails and unstable circulation remains.

Regarding what measures should be taken by young physicians in a standard hospital without a hybrid ER to obtain a survival outcome in patients with fatal azygos injury and unstable circulation until veteran surgeons arrive. Aside from the ATLS® protocol, a 1:1:1 ratio of packed red blood cells, fresh plasma, and platelets with minimal crystalloids is the preferred resuscitative strategy to avoid diluted coagulopathy by crystalloid fluid resuscitation^42)^. Recently, in patients experiencing hemorrhagic shock, whole-blood transfusion was reported to be associated with both an improved survival and decreased overall blood utilization^43)^. If a patient does not obtain stable circulation even after massive transfusion, they should be intubated to secure the airway^35)^. After definitively securing the airway, a CT examination should be considered, although the proper timing of CT remains controversial^44)^. A chest drain is usually inserted to drain the hemothorax and evaluate the volume in order to decide the timing of radical operation. Tentative drain clamping may be effective for achieving hemostasis at the bleeding source or reducing the total hemorrhaging volume by the hematoma tamponade effect, based on our personal experience and evidence from total knee arthroplasty^45)^. However, it should be noted that drain clamping may result in hemorrhagic death or fatal tension hemothorax. Intensive hypotensive resuscitation is recommended, as it is safe and has a lower mortality rate than normotensive resuscitation in hemorrhagic shock patients. There is also less blood loss, hemodilution, ischemia, and hypoxia in tissues with such an approach^46)^. If young physicians aggressively attempt damage control intervention using right thoracotomy but fail to identify the bleeding source, hilar clamping or twisting may be attempted to detect the bleeding source^47, 48)^. If the bleeding cannot be stopped with these procedures, the bleeding source likely lies outside of the pulmonary artery and venous system. In addition, in cases with an unknown bleeding source, a large amount of gauze should be packed blindly in order to achieve hemostasis^49)^. Alternatively, clam-shell thoracotomy may be useful for identifying the bleeding source, even in the supine position^50)^.

## Conclusion

We presented a fatal case of blunt azygos injury and the results of an analysis of the relevant literature. ER physicians must consider azygos vein injury as a possible cause of right hemothorax in patients with blunt chest trauma if the individual shows persistent hypotension. In addition, the early recognition of the site of hemorrhaging using CT may be required, even if the patient's circulation remains unstable after initial fluid resuscitation.

## Funding

This work was supported in part by a Grant-in-Aid for Special Research in Subsidies for ordinary expenses of private schools from The Promotion and Mutual Aid Corporation for Private Schools of Japan.

## Author contributions

KM was a major contributor in writing the manuscript. KJ, SH and YY were editing the manuscript. All authors read and approved the final manuscript.

## Conflicts of interest statement

We do not have conflict of interest to declare.

## References

[1] DeMaio K, Kaushik S, Vadlamudi V: Endovascular treatment of traumatic azygous vein injuries: a case report. CVIR Endovascular,, 2021; 4: 48. 34097160 10.1186/s42155-021-00235-5 PMC8184975

[2] Li C, Martin K, Reada D: Azygos vein lacerations, a rare injury from high-impact chest trauma: Two cases and a review of the literature. Int J Surg Case Rep, 2022; 91: 106778. 35051887 10.1016/j.ijscr.2022.106778 PMC8858728

[3] Laohathai S: An isolated azygos vein rupture from blunt chest trauma. Asian Cardiovasc Thorac Ann, 2019; 27: 757-759. 31072105 10.1177/0218492319850049

[4] Papadomanolakis A, Theodoridou E, Vogiatzis N, Pentheroudaki A, Daskalaki D, Lolis ED: Injury to Azygos Venous System: A Co-Existing Injury in High-Impact Lethal Trauma. World J Surg, 2016; 40: 1355-61. 26817649 10.1007/s00268-016-3411-7

[5] Yang E, Jeong W, Lee J, Kim S: Life-threatening hemothorax due to azygos vein rupture after chest compression during cardiopulmonary resuscitation. Am J Emerg Med, 2014; 32: 1437.e1-2. 10.1016/j.ajem.2014.04.019 24881516

[6] Mohajeri G, Hekmatnia A, Ahrar H, Hekmatnia F, Basiratnia R: Azygos vein aneurysm as a posterior mediastinal mass discovered after minor chest trauma. Iran J Radiol, 2014; 11: e7467. 24693303 10.5812/iranjradiol.7467 PMC3955859

[7] Haq AA, Restrepo CS, Lamus D, Ocazionez-Trujillo D, Vargas D: Thoracic venous injuries: an imaging and management overview. EmergRadiol, 2016; 23: 291-301. 10.1007/s10140-016-1386-1 26965007

[8] Cao JG, Dai NF, Chen CZ: Ruptured azygos vein caused by blunt trauma on left chest. Chin Med J (Engl), 2012; 125: 3355-6. 22964338

[9] Juraszyński Z, Zieliński D, Burakowska B: Trivial chest injury leading to azygos vein pseudoaneurysm. Am J Med, 2010; 123: e9-e10. 10.1016/j.amjmed.2010.03.032 20920683

[10] Endara SA, Davalos GA, Nuñez MF, Manzano JE: Azygous vein laceration secondary to blunt thoraco-abdominal trauma. Interact Cardiovasc Thorac Surg, 2010; 11: 342-4. 20576652 10.1510/icvts.2010.234666

[11] Drac P, Manak P, Klein J, Kral V: Azygos vein injury in blunt chest trauma. Biomed Pap Med Fac Univ Palacky Olomouc Czech Repub, 2007; 151: 347-8. 18345276 10.5507/bp.2007.059

[12] Kamiyoshihara M, Ibe T, Kakegawa S: Blunt traumatic injury of the azygous vein diagnosed by computed tomography. Eur J Cardiothorac Surg, 2007; 31: 307. 17174560 10.1016/j.ejcts.2006.11.022

[13] Nguyen LL, Gates JD: Simultaneous azygous vein and aortic injury from blunt trauma: Case report and review of the literature. J Trauma, 2006; 61: 444-6. 16917464 10.1097/01.ta.0000229959.54552.be

[14] Bowles BJ, Teruya T, Belzberg H, Rivkind AI: Blunt traumatic azygous vein injury diagnosed by computed tomography: case report and review of the literature. J Trauma, 2000; 49: 776-9. 11038104 10.1097/00005373-200010000-00034

[15] Sharma OP, Rawitscher RE: Blunt vena azygos trauma: report of a case and review of world literature. J Trauma, 1999; 46: 192-5. 9932707 10.1097/00005373-199901000-00034

[16] Sugimoto K, Asari Y, Hirata M, Imai H, Ohwada T: The diagnostic problem associated with blunt traumatic azygous vein injury: delayed appearance of right haemothorax after blunt chest trauma. Injury, 1998; 29: 380-2. 9813684 10.1016/s0020-1383(97)00173-3

[17] Cagini L, Boaron M, Corneli G, et al: Rupture of the azygos vein in blunt chest trauma. J Cardiovasc Surg (Torino), 1998; 39: 249-50. 9639015

[18] [No authors listed]. Image interpretation session: 1995. Case 3. Posttraumatic pseudoaneurysm of the azygos vein in a patient with azygos continuation of the IVC. Radiographics, 1996; 16: 220-2. 10946705 10.1148/radiographics.16.1.220

[19] Butler DA, Schneider RF, Jadali M: Traumatic injury to the azygous vein: case report. J Trauma, 1995; 39: 761-2. 7473972 10.1097/00005373-199510000-00029

[20] Jain A, Blebea JS: Post-traumatic pseudoaneurysm of the azygous vein in a patient with azygous continuation. J Comput Assist Tomogr, 1994; 18: 647-8. 8040455 10.1097/00004728-199407000-00026

[21] Inoue H, Iwasaki M, Shirota S, Ogawa J, Shohtsu A: Total avulsion of the azygos vein and longitudinal laceration of the mediastinal pleura due to blunt chest trauma: a case report. J Cardiovasc Surg (Torino), 1993; 34: 67-8. 8482708

[22] Walsh A, Snyder HS: Azygos vein laceration following a vertical deceleration injury. J Emerg Med, 1992; 10: 35-7. 1629589 10.1016/0736-4679(92)90008-h

[23] Thurman RT, Roettger R: Intrapleural rupture of the azygos vein. Ann Thorac Surg, 1992; 53: 697-9. 1554286 10.1016/0003-4975(92)90339-6

[24] Shkrum MJ, Green RN, Shum DT: Azygos vein laceration due to blunt trauma. J Forensic Sci, 1991; 36: 410-21. 2066722

[25] Baldwin JC, Oyer PE, Guthaner DF, Stinson EB: Combined azygous vein and subclavian artery injury in blunt chest trauma. J Trauma, 1984; 24: 170-1. 6694246 10.1097/00005373-198402000-00018

[26] Sherani TM, Fitzpatrick GJ, Phelan DM, O’Brien D, Tarief HA, Neligan MC: Ruptured azygos vein due to blunt chest trauma. Br J Surg, 1986; 73: 885. 3790913 10.1002/bjs.1800731109

[27] Coates GR, Hall DP: Rupture of the azygos vein: an unusual cause of hemothorax due to blunt trauma. J Tenn Med Assoc, 1987; 80: 155-6. 3560929

[28] Snyder CL, Eyer SD: Blunt chest trauma with transection of the azygos vein: case report. J Trauma, 1989; 29: 889-90. 2661848 10.1097/00005373-198906000-00033

[29] Wall MJ Jr, Mattox KL, Debakey ME: Injuries to the azygous venous system. J Trauma, 2006; 60: 357-62. 16508496 10.1097/01.ta.0000202552.26628.84

[30] Tran A, Yates J, Lau A, Lampron J, Matar M: Permissive hypotension versus conventional resuscitation strategies in adult trauma patients with hemorrhagic shock: A systematic review and meta-analysis of randomized controlled trials. J Trauma Acute Care Surg, 2018; 84: 802-808. 29370058 10.1097/TA.0000000000001816

[31] Gupta A, Kumar S, Sagar S, et al: Damage control surgery: 6 years of experience at a level I trauma center. Ulus Travma Acil Cerrahi Derg, 2017; 23: 322-327. 28762453 10.5505/tjtes.2016.03693

[32] Tiel Groenestege-Kreb D, van Maarseveen O, Leenen L: Trauma team. Br J Anaesth, 2014; 113: 258-65. 24980423 10.1093/bja/aeu236

[33] Li H, Lin J, Zhang H, et al: A propensity score matching study of the short-term efficacy of azygos arch-sparing McKeown minimally invasive esophagectomy. J Gastrointest Oncol, 2021; 12: 28-37. 33708422 10.21037/jgo-21-14 PMC7944159

[34] Morrison JJ, Mellor A, Midwinter M, Mahoney PF, Clasper JC: Is pre-hospital thoracotomy necessary in the military environment? Injury, 2011; 42: 469-73. 20362287 10.1016/j.injury.2010.03.009

[35] Galvagno SM Jr, Nahmias JT, Young DA: Advanced Trauma Life Support Update 2019: Management and Applications for Adults and Special Populations. Anesthesiol Clin, 2019; 37: 13-32. 30711226 10.1016/j.anclin.2018.09.009

[36] Motomura T, Mashiko K, Matsumoto H, et al: Preventable trauma deaths after traffic accidents in Chiba Prefecture, Japan, 2011: problems and solutions. J Nippon Med Sch, 2014; 81: 320-7. 25391701 10.1272/jnms.81.320

[37] Dub L, Thomas SZ, Fusco N, Plamoottil CI, Ganti L: A Rapid Diagnosis and Treatment of a Traumatic Aortic Transection: A Case of Survival to the ICU. Cureus, 2021; 13: e12726. 33614329 10.7759/cureus.12726 PMC7883568

[38] Kinoshita T, Yamakawa K, Matsuda H, et al: The Survival Benefit of a Novel Trauma Workflow that Includes Immediate Whole-body Computed Tomography, Surgery, and Interventional Radiology, All in One Trauma Resuscitation Room: A Retrospective Historical Control Study. Ann Surg, 2019; 269: 370-376. 28953551 10.1097/SLA.0000000000002527 PMC6325752

[39] Founding members of the Japanese Association for Hybrid Emergency Room System (JA‐HERS): The hybrid emergency room system: a novel trauma evaluation and care system created in Japan. Acute Med Surg, 2019; 6: 247-251. 31304025 10.1002/ams2.412 PMC6603312

[40] Kinoshita T, Moriwaki K, Hanaki N, et al: Cost-effectiveness of a hybrid emergency room system for severe trauma: a health technology assessment from the perspective of the third-party payer in Japan. World J Emerg Surg, 2021; 16: 2. 33413503 10.1186/s13017-020-00344-x PMC7791815

[41] Umemura Y, Watanabe A, Kinoshita T, Morita N, Yamakawa K, Fujimi S: Hybrid emergency room shows maximum effect on trauma resuscitation when used in patients with higher severity. J Trauma Acute Care Surg, 2021; 90: 232-239. 33165282 10.1097/TA.0000000000003020

[42] Black JA, Pierce VS, Juneja K, Holcomb JB: Complications of Hemorrhagic Shock and Massive Transfusion-a Comparison Before and After the Damage Control Resuscitation Era. Shock, 2021; 56: 42-51. 10.1097/SHK.0000000000001676 34196627

[43] Brill JB, Tang B, Hatton G, et al: Impact of Incorporating Whole Blood into Hemorrhagic Shock Resuscitation: Analysis of 1,377 Consecutive Trauma Patients Receiving Emergency-Release Uncrossmatched Blood Products. J Am Coll Surg, 2022; 234: 408-418. 35290259 10.1097/XCS.0000000000000086

[44] Murao S, Yamakawa K, Kabata D, et al: Effect of Earlier Door-to-CT and Door-to-Bleeding Control in Severe Blunt Trauma: A Retrospective Cohort Study. J Clin Med, 2021; 10: 1522. 33917338 10.3390/jcm10071522 PMC8038745

[45] Hishimura R, Onodera T, Ohkoshi Y, Okada K, Matsuoka M, Matsubara S, Iwasaki K, Kondo E, Iwasaki N: The effect of local injection of tranexamic acid into peri-articular tissue versus drain clamping in total knee arthroplasty: a randomized controlled trial. BMC Musculoskelet Disord, 2022; 23: 111. 35109837 10.1186/s12891-022-05058-6 PMC8808990

[46] Woodward L, Alsabri M: Permissive Hypotension vs. Conventional Resuscitation in Patients With Trauma or Hemorrhagic Shock: A Review. Cureus, 2021; 13: e16487. 34430103 10.7759/cureus.16487 PMC8372825

[47] Garcia A, Martinez J, Rodriguez J, et al: Damage-control techniques in the management of severe lung trauma. J Trauma Acute Care Surg, 2015; 78: 45-50. 25539202 10.1097/TA.0000000000000482 PMC4279445

[48] Wilson A, Wall MJ Jr, Maxson R, Mattox K: The pulmonary hilum twist as a thoracic damage control procedure. Am J Surg, 2003; 186: 49-52. 12842749 10.1016/s0002-9610(03)00102-8

[49] Moriwaki Y, Toyoda H, Harunari N, Iwashita M, Kosuge T, Arata S, Suzuki N: Gauze packing as damage control for uncontrollable haemorrhage in severe thoracic trauma. Ann R Coll Surg Engl, 2013; 95: 20-5. 23317720 10.1308/003588413X13511609956057 PMC3964630

[50] Katiyar A, Kumar A, Kumar N, Kataria R, Sarkar B: A successful off-pump cardiac repair following blunt cardiac rupture - A case report. Trauma Case Rep, 2020; 29: 100344. 32875048 10.1016/j.tcr.2020.100344 PMC7451814

